# *Burkholderia pseudomallei* and Melioidosis in Mainland Southeast Asia: A Regional Synthesis of Ecological, Epidemiological, and Public Health Perspectives

**DOI:** 10.1155/tbed/5555905

**Published:** 2026-10-28

**Authors:** Kimyeun Oeurn, Vincent Herbreteau, Sokleaph Cheng, Thi Thu Hang Le, Anne-Laure Bañuls, Mallorie Hide

**Affiliations:** ^1^ Department of Life Sciences University of Science and Technology of Hanoi Hanoi Vietnam usth.edu.vn; ^2^ Bacteriology and Antibiotic Resistance Group, Medical Biology Laboratory Institut Pasteur du Cambodge, Pasteur Network Phnom Penh Cambodia; ^3^ LMI Drug Resistance In Southeast Asia IRD Montpellier France ird.fr; ^4^ MIVEGEC CNRS IRD Montpellier University Montpellier France umontpellier.fr; ^5^ ESPACE-DEV IRD Univ Reunion Univ Guyane Univ Montpellier Montpellier France umontpellier.fr; ^6^ Khmer Earth OBServation (KHEOBS) Laboratory Institute of Technology of Cambodia Phnom Penh Cambodia

**Keywords:** animal, *Burkholderia pseudomallei*, environment, human, Mainland Southeast Asia, melioidosis

## Abstract

*Burkholderia pseudomallei* (*B. pseudomallei*), the causative agent of melioidosis, is endemic to tropical and subtropical regions, particularly in Mainland Southeast Asia (MSEA). Despite its significant impact on human and animal health, the ecological distribution and public health burden of this environmental pathogen are still poorly documented. In MSEA, the bacterium is widespread, especially in rice–paddy irrigation water, canals, rivers, waterfalls, drinking‐water sources, and even in ornamental fish tanks. It has been detected at various soil depths, up to 3 m, and is commonly associated with specific soil types. Environmental factors, including the physicochemical properties of soil and water, strongly influence the distribution of *B. pseudomallei*. Natural disasters and extreme weather events such as flooding, tsunamis, and heavy rainfall can significantly increase the incidence of melioidosis in MSEA. Animal infections have been reported, including outbreaks in zoos and domestic animals across MSEA. Despite its public health impact, melioidosis remains a non‐notifiable disease in several MSEA countries, including Cambodia, Lao PDR, Myanmar, Peninsular Malaysia, and Vietnam. Treatment guidelines have been developed in several MSEA countries, although national guidance is absent or incomplete in Myanmar and is available only at the state level in Peninsular Malaysia. *B. pseudomallei* is widely distributed throughout the environment in MSEA, highlighting the need for integrated surveillance of environment, animal, and human infection. This narrative review aims to provide a comprehensive overview of the current knowledge on the ecology, epidemiology, and public health aspects of *B. pseudomallei* and melioidosis in MSEA, highlighting key knowledge gaps and priorities for surveillance, laboratory capacity, and cross‐border collaboration. Understanding the environmental distribution of *B. pseudomallei* and its links to climate and ecological factors is essential for anticipating changes in disease risk and implementing effective One Health strategies across MSEA.

## 1. Introduction

Melioidosis is a tropical infectious disease caused by the environmental bacterium *Burkholderia pseudomallei* (*B. pseudomallei*). Within the genus *Burkholderia*, which contains over 80 formally named species, *B. pseudomallei* is an aerobic, nonspore‐forming, nonfermenting, Gram‐negative bacillus [1]. *B. pseudomallei* is classified as a Tier 1 select agent by the Centers for Disease Control and Prevention in the USA. It is frequently found in the rhizosphere, moist soil, and both surface water and groundwater [1]. Patients with melioidosis experience high rates of bacteremia, organ failure, and death [2]. Melioidosis presents with a wide spectrum of clinical manifestations, ranging from mild localized infection to severe sepsis [3]. Transmission of *B. pseudomallei* to animals and humans occurs through cutaneous entry, ingestion, or inhalation of contaminated soil or water (Figure 1). In humans, cases of transmission of melioidosis from mother to infant through breast milk in the event of mastitis have been reported [4]. In general, vertical and sexual transmission, zoonotic transmission, nosocomial infection, and transmission to laboratory staff are rarely reported [4]. In 2015, a comprehensive global modeling estimated that there were 165,000 human melioidosis cases and 89,000 deaths worldwide per year, primarily in low‐ and middle‐income countries, due to challenges in the diagnosis and ineffective antimicrobial treatments [5]. At that time, 45 countries were known to be endemic of melioidosis (in Latin and South America, in Africa, and in Asia), and 34 countries (mostly in Sub‐Saharan Africa) were probably endemic but never reported [5]. A recent review of the global distribution of *B. pseudomallei* based on consensus evidence from January 1910 to September 2022 included its presence in 12 new countries, and for the first time, in the southern United States [6]. Melioidosis has been called on to be recognized as a neglected tropical disease (NTD) by WHO [7].

**Figure 1:**
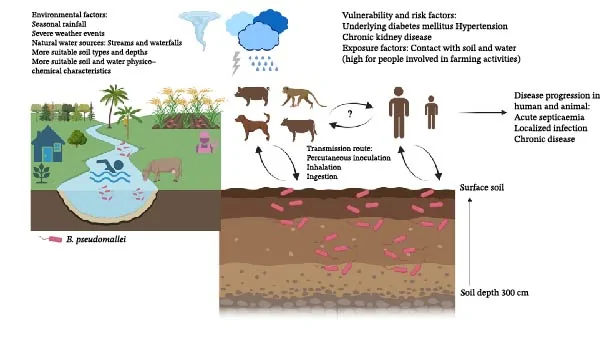
The transmission life cycle of *B. pseudomallei* in Mainland Southeast Asia (MSEA), figure created using BioRender.com.

The aim of this narrative review is to provide an overview of the current state of knowledge regarding *B. pseudomallei* and melioidosis in Mainland Southeast Asia (MSEA) (Cambodia, Lao PDR, Myanmar, Peninsular Malaysia, Thailand, and Vietnam), where previous global reviews have shown a high endemicity and where melioidosis remains underdiagnosed.

## 2. Methodology

The narrative literature review was conducted through PubMed and Scopus databases from inception to December 29, 2025, using lists of keywords referring to the medical subject headings (MeSH) thesaurus. These keywords were combined using the Boolean operators OR (within key concepts) and AND (between key concepts) as follows: (“melioidosis” OR “*Burkholderia pseudomallei*”) AND (“Cambodia”). A similar search strategy was applied to Laos (OR Lao PDR), Myanmar, Peninsular Malaysia, Thailand, and Vietnam. Other keywords such as “water,” “soil,” “environment,” “treatment,” “seroprevalence/serology,” “animal,” “diagnosis,” “detection,” and “climatic/climate” were added to refine the search when needed. In addition to the electronic database search, reference lists of included articles and relevant review papers were manually screened to identify additional eligible studies. Researchers in the field of melioidosis were consulted to identify unpublished or ongoing studies relevant to this review, including national treatment guidelines from Thailand, Vietnam, and Myanmar and unpublished data from the WatHealth project in Cambodia. We first screened the titles and abstracts of identified publications, followed by a full‐text assessment based on the predefined eligibility criteria. Studies were included if they were original research articles reporting data from any of the six MSEA countries and addressing at least one of the following topics: human, animal, or environmental compartment of *B. pseudomallei*, climatic factors, or antimicrobial resistance (AMR). Eligible study designs included case reports, case series, observational studies, outbreak investigations, environmental studies, laboratory studies, diagnostic studies, and molecular epidemiological studies. Articles published in English as well as one national treatment guideline in Thai and two articles in French were included. Experimental animal and cell line studies, clinical trials, and studies exclusively addressing the radiological features of melioidosis were excluded. A preferred reporting items for systematic reviews and meta‐analyses (PRISMA)‐style flow diagram illustrating the study identification and selection process is presented in Figure 2.

**Figure 2:**
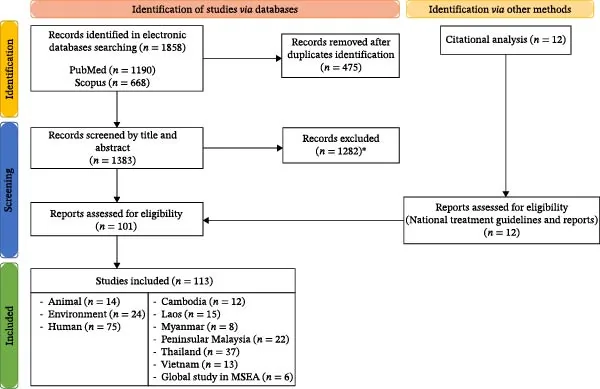
PRISMA‐style flow diagram of the study identification and selection process.  ^∗^Records were excluded according to the predefined eligibility and exclusion criteria described in the Methods section.

## 3. *B. pseudomallei* in the Environment

*B. pseudomallei* is an environmental bacterium commonly found in soil and surface water in tropical and subtropical regions, where its long‐term persistence could facilitate accidental infections in humans, usually through contact with contaminated soil or water. This first part will therefore focus on the environmental compartment.

### 3.1. *B. pseudomallei* in Soil

*B. pseudomallei* is widely distributed in the environment, particularly in soil and water throughout MSEA, with detection rates and concentrations varying among the six countries [8–13]. In rice fields, environmental studies have reported a median count of 700 colony‐forming units per gram (CFU/g) in northeastern Thailand [14], 90 CFU/g in Siem Reap Province, Cambodia [13], and 39 CFU/g in the Vientiane region of Lao PDR [15]. A large‐scale study analyzing 6100 soil samples from 61 rice fields across eastern, central, and northeastern Thailand detected *B. pseudomallei* in 49% of the fields (30 out of 61) [16]. Although these findings suggest geographical variation, direct comparisons between countries are limited by considerable differences in study design, environmental sampling protocols, and laboratory methodologies, which may contribute to the observed variability. Despite these limitations, the available evidence consistently suggests that land use plays an important role in shaping the environmental distribution of *B. pseudomallei*. Spatial analyses in Kedah, Peninsular Malaysia, demonstrated strong associations between melioidosis risk and land cover types [17]. These included areas dominated by large‐scale irrigation agriculture, mixed agriculture and pastoralism, urban settings, and various forestry combinations. The findings suggested that areas with greater human modification of natural landscapes may be associated with an increased melioidosis risk [17]. Similarly, a broader survey of 3870 soil samples across Myanmar’s states and regions found that isolation rates were higher in agricultural (2.2%), pasture (8.5%), and disused lands (5.8%) compared to residential areas (0.4%), though these differences were not statistically significant [18]. In the northern and north‐central regions of Vietnam, the bacterium was detected in soil from rice fields and residential gardens [11, 19]. Taken together, these studies indicate that *B. pseudomallei* occupies diverse environmental habitats across MSEA. Although agricultural land use is a factor consistently associated with bacterial detection, the strength of this association varies between studies, highlighting that local ecological conditions and methodological differences also contribute to the observed spatial patterns.

Soil characteristics appear to influence the ecological niche of *B. pseudomallei*. Sandy soils, which facilitate bacterial movement through water flow, also support the survival of *B. pseudomallei*. In northeastern Thailand, 76% of soil samples were classified as sandy, and more than half (54.2%) of these samples tested positive for *B. pseudomallei* [20]. Likewise, sandy soils (6.3%) in Myanmar showed higher positivity than other soil types [18]. Beyond soil texture, several studies have explored the influence of reference soil groups (RSGs) including acrisols, alisols, luvisols, and anthrosols, which are the first‐level categories of the World Reference Base for Soil Resources, grouping the major soils of the world according to diagnostic horizons, properties, and materials [21]. In Lao PDR, associations between *B. pseudomallei* and specific soil types (with clay‐enriched subsoil) have been well established. In Saravane Province, 94% of positive samples in the east were linked to ferric alisols (high‐activity clays, low base status), while the west and center showed associations with 61% ferric acrisols (low‐activity clays, low base status) and 42% ferric luvisols (high‐activity clays, high base status), respectively [22]. Ribolzi et al. [23] showed that positive qPCR to *B. pseudomallei* detection were positively associated with acrisol and luvisol soil types. Limmathurotsakul et al. [5] predicted the global distribution of *B. pseudomallei*, and they found that anthrosol (with long and intensive agricultural use) and acrisol soil types were strongly associated with the presence of *B. pseudomallei*. According to calculations using the Digital Soil Map of the World (to classify soils and differentiates areas by different soil units) by the FAO (2007), acrisols cover over 50.4% of MSEA, with Lao PDR having more than 70% coverage, mainly in river valleys, lowland plains, and areas along major rivers [24]. Although these observations suggest that certain soil groups provide favorable habitats for *B. pseudomallei*, soil classification alone is unlikely to determine bacterial occurrence. Rather, these soil types probably represent environments with physicochemical and hydrological characteristics that collectively promote bacterial survival.

The bacterium *B. pseudomallei* is able to survive at different depths within the soil environment. In Myanmar, *B. pseudomallei* was isolated from rice‐farming areas and disused land at soil depths of 30, 60, and 90 cm, although with a low detection rate of 1% (4 out of 400 samples) [12]. Additionally, soils collected at 30 cm depth from 32 small ruminant farms in Peninsular Malaysia have also been tested positive for *B. pseudomallei* [25]. Another study in Lao PDR, investigating gleyic plinthic acrisols from the surface to 300 cm found the highest bacterial concentrations between 110 and 250 cm depth [26]. Overall, the presence of *B. pseudomallei* has been detected in all MSEA countries with an association with several RSGs (acrisols, alisols, luvisols, and anthrosols). However, these associations are observational and do not imply that soil type alone determines bacterial distribution. The bacterium also occurs at considerable soil depths, suggesting that deeper soil layers may provide favorable conditions for persistence. Nevertheless, methodological differences in sampling depth and study design limit direct comparisons, and the ecological importance of vertical distribution remains poorly understood. In addition to land use, soil type, and soil depth, the persistence of *B. pseudomallei* is influenced by a range of physicochemical properties. The association between the bacteria and a range of soil physicochemical characteristics is heterogeneous across studies and environmental settings (Table 1). In Thailand, bacterial isolation was associated with moderately acidic soils, higher moisture content, increased sand content, and elevated chemical oxygen demand (COD) and total nitrogen, whereas several other soil properties showed negative associations [16, 20]. However, studies from Peninsular Malaysia and Lao PDR identified different environmental correlates, including higher iron, clay, and moisture content in Peninsular Malaysia [25], and lower concentrations of nitrogen, organic carbon, and organic matter in Lao PDR [29]. Additionally, a deeper soil analysis extending to 300 cm revealed that the distribution of *B. pseudomallei* was closely linked to variations in physico‐chemical properties at different depths [26]. Although several soil physicochemical properties have been associated with *B. pseudomallei*, the reported relationships are not consistent across studies, indicating that these factors are context‐dependent rather than universal predictors of bacterial occurrence.

**Table 1:** Physico‐chemical characteristics of soil and water influencing the occurrence of *B. pseudomallei* in MSEA.

Country	Physico‐chemical properties	Environment	Reference
Lao PDR	Positive correlation: GPR, Ks, Fe, K, Al, Mg, Clay, Poro, Ti, Slit, WC, K_e, Mg_e, and Ca_e Negative correlation: low pH (median 4.86), Ca, Si, Sand, and BD	Soil (along 300 cm)	[26]
Lao PDR	Low pH (median 7.62), low levels of lined drain cement, high turbidity and TDS	Water	[27, 28]
Lao PDR and Peninsular Malaysia	High in Fe, clay and water; low in N, Org. C, and O.M.	Soil (30 cm); (5, 30, 60 and 90 cm)	[25, 29]
Peninsular Malaysia	Slightly acidic water pH (pH = 6.21) and high COD	Water	[9]
Thailand	pH = 5.6, high levels of moisture content N and COD	Soil (30 cm)	[20]
Thailand	Positive correlation (high level): the proposition of sand Negative correlation (low level): clay, moisture, salinity, O.M., Cd, cation exchange capacity, carbon to nitrogen ratio, and nutrients (P, K, Ca, Mg, and Fe)	Soil (30 cm)	[16]

*Note:* Ks, Saturated Hydraulic Conductivity; Poro, Soil Porosity.

Abbreviations: Al, Aluminium; BD, bulk density; Ca, calcium; Cd, cadmium; COD, chemical oxygen demand; Fe, iron; GPR, groundwater persistence rate; K, potassium; K_e, Mg_e and Ca_e, exchangeable potassium, magnesium, and calcium; Mg, magnesium; N, nitrogen; O.M., organic matter; Org. C, organic carbon; P, phosphorus; Si, silicium; TDS, total dissolved solids; Ti, titanium; WC, water content.

### 3.2. *B. pseudomallei* in Water

*B. pseudomallei* is not only present in soil but is frequently detected in a variety of environmental waters across MSEA, including rice–paddy irrigation water, canals, rivers, waterfalls, drinking‐water sources, and even in ornamental fish tanks. This highlights the fact that environmental water represents an important reservoir of potential human exposure. The organism was found in multiple water bodies, such as ponds, rainwater jars, dug wells, and pump well jars. These sources were used for a variety of domestic and recreational purposes, including drinking, cooking, bathing, feeding livestock, and leisure activities by affected patients and their families in Buriram Province (northeastern Thailand) [30].

Evidence from across MSEA indicates that the occurrence of *B. pseudomallei* in water is highly variable, reflecting not only differences in local environmental and seasonal conditions but also variations in sampling strategies and laboratory detection methods. For example, a recent study in the Kandal province of Cambodia has highlighted the presence of *B. pseudomallei* in canal and rice‐field water using both qPCR detection and microbial isolation (Hide M et al., unpublished data). Similarly, in north‐central Vietnam (Thanh Hoa, Nghe An, and Ha Tinh provinces), *B. pseudomallei* was detected in 43.6% (349/800) of paddy field water samples by TTSS1‐targeted realtime PCR and isolation on Ashdown agar [19]. The culture positivity rate for *B. pseudomallei* varied markedly by season, ranging from as low as 5% in winter to as high as 82% during the summer. Moreover, the bacterium showed a highly heterogeneous distribution in paddy field water, varying both spatially and temporally, even within a single small rice field [19]. Beyond surface waters in rice fields and canals, *B. pseudomallei* has also been isolated from water supplies on small ruminant farms in Peninsular Malaysia and borehole water in northern Vietnam [9, 11], suggesting that potential exposure extends beyond agricultural irrigation systems. In contrast, irrigation water on farms in Myanmar has been investigated, but the bacteria has not been detected in the water source, possibly reflecting the limited number of environmental studies conducted there [31].

River systems may facilitate the environmental distribution of *B. pseudomallei*. In Lao PDR, river water was more likely to be contaminated with *B. pseudomallei* than water from rice fields [27]. The bacterium was detected in 9% of river samples during the dry season and in 57% during the rainy season, as well as in 33.3% of drainage water samples during the rainy season [28, 32]. These findings indicate substantial environmental variability and suggest that rivers may serve as useful environmental sentinels for *B. pseudomallei*. They also suggest that exposure in urban Vientiane may be more widespread than previously estimated.

The natural water sources, including streams and waterfalls, were frequently found to contain *B. pseudomallei*, with waterborne exposure identified as an important route of melioidosis in Songkhla Province, the second largest province in southern Thailand [33]. A recent outbreak of *B. pseudomallei* occurred in nonpotable tap water in Saraburi Province, located in central Thailand, due to inadequate maintenance of the water chlorination system. Patients affected by the outbreak reported using nonpotable tap water for everyday activities such as brushing teeth, rinsing the mouth, and showering [34]. Furthermore, *B. pseudomallei* has been detected in drinking water sources in several regions of Thailand, including Ubon Ratchathani Province in the Northeast and Koh Phangan, an island in the Gulf of Thailand in the South [35–37]. Ornamental fish tanks were more likely to be contaminated with *B. pseudomallei* in Vientiane, the capital of Lao PDR [38]. These findings indicate that exposure to *B. pseudomallei* is not limited to natural water bodies but may also occur through treated and domestic water systems when contamination control is inadequate.

In MSEA, several water parameters have also been associated with the occurrence of *B. pseudomallei*, including a pH range of 6.21–7.62, high levels of COD, turbidity, and total dissolved solids (TDS), as well as reduced levels of lined drain cement (Table 1) [9, 27, 28]. Taken together, these findings indicate that *B. pseudomallei* is widely distributed in aquatic environments across the MSEA, but the environmental factors associated with its occurrence vary considerably between studies and locations.

### 3.3. Climatic Parameters Associated With *B. pseudomallei* in the Environment

Natural disasters and extreme weather events have been linked to an increased incidence of melioidosis. A study conducted in Cambodia and Lao PDR reported a significant seasonal burden of melioidosis among both adults and children, linked to increased humidity and wind speed [39]. The association between rainfall and melioidosis incidence has been well documented in Cambodia, Peninsular Malaysia, and Thailand [39–41]. Similarly, cases often surge following flooding. A field study conducted in January 2015 across eight districts of Kelantan State, Peninsular Malaysia, 2 weeks after a major flood, found that *B. pseudomallei* was present in 1% of soil samples, suggesting a potential health risk for flood‐affected populations [42]. In Pahang State, 10 cases of melioidosis were reported in association with search and rescue operations conducted in a natural recreational forest featuring a waterfall and stream. Of these, six were melioidosis‐only infections, while four were co‐infected with leptospirosis. Notably, eight fatalities occurred during the rescue efforts for drowning victims, underscoring the serious health risks linked to environmental exposure to *B. pseudomallei* during natural disasters [43]. In southern Thailand, a noticeable rise in melioidosis cases during 2016–2017 was associated with two major flood events occurring at the end of each year [40]. In central Thailand, a region not typically endemic for melioidosis, five cases were reported within 4 weeks of severe flooding during the rainy season [44]. Following the tsunami on December 26, 2004, which severely affected southern Thailand, there was a marked increase in melioidosis cases, with six cases occurring in 1 year compared with nine cases over the previous 6 years [45]. A recent study from north‐central Vietnam found that higher ambient temperatures significantly increased both the occurrence and bacterial load of *B. pseudomallei* in surface water. When combined with heavy rainfall or agricultural occupational exposure, these conditions may contribute to a higher risk of melioidosis [19]. Collectively, the available evidence supports that extreme weather events can amplify exposure to *B. pseudomallei*, but further longitudinal environmental and epidemiological studies are needed to clarify the mechanisms linking climatic variability to melioidosis transmission across the MSEA.

## 4. *B. pseudomallei* in Animals

A wide variety of animals can be infected with *B. pseudomallei*, including terrestrial and aquatic mammals, birds, fish, and domestic animals, with the severity and clinical manifestations varying across species [46]. Humans and animals typically contract the disease through similar routes of exposure [46]. In animals, melioidosis can present as acute septicemia, localized infection, or chronic disease. Notably, most zoo animals succumb rapidly following the onset of clinical signs, whereas chronic infections are more commonly observed in domestic species such as cattle, pigs, dogs, and cats [47]. Cases of animal melioidosis have been reported across MSEA.

### 4.1. Cambodia

No cases of *B. pseudomallei* infections have been reported in animals in Cambodia. However, since 2020, the organism has been detected in cynomolgus macaques (*Macaca fascicularis*) that were imported into the United States from Cambodia. This represents the first documented case of melioidosis identified during the CDC‐mandated post‐import quarantine period [48]. Although no indigenous animal cases have been reported, the detection of *B. pseudomallei* in exported cynomolgus macaques suggests that animal infections may be under‐recognized in Cambodia.

### 4.2. Lao PDR

A case of melioidosis in a goat was first confirmed in 2003. By 2025, a livestock survey across 16 provinces had identified significant clusters of animals exposed to *B. pseudomallei* with the highest seroprevalence found in cattle (22.8%), followed by buffalo (19.7%), and pigs (4.0%) [49, 50]. These findings indicate widespread exposure of livestock to *B. pseudomallei*, suggesting that the bacterium is broadly distributed across Lao PDR.

### 4.3. Myanmar

To date, no cases of melioidosis have been diagnosed in animals in Myanmar [51]. The absence of reported cases is more likely to reflect limited veterinary investigations and diagnostic capacity than the absence of *B. pseudomallei* in animal populations.

### 4.4. Peninsular Malaysia

In Malaysia, melioidosis has been reported in a wide range of animals, including goats, sheep, cattle, pigs, deer, monkeys, horses, cats, dogs, rabbits, and primates [52]. Data from continuous surveillance and monitoring of melioidosis in animals over a 10‐year period (2000–2009) were obtained from the Department of Veterinary Services and the Veterinary Research Institute. Surveillance was conducted throughout Malaysia and included 11 states in Peninsular Malaysia. The results revealed the presence of antibodies against *B. pseudomallei* among different livestock species, with rates of 7.6% in cattle, 48.2% in buffalo, 2.6% in goats, 13.6% in sheep, and 3.6% in pigs. Annual seroprevalence ranged from a low of 3.3% in 2008 to a high of 12.0% in 2003. The mean yearly seroprevalence over the study period was 6.9%, while the overall seroprevalence was 6.1% [53]. Data from passive surveillance of melioidosis conducted at the Serology Laboratory of the Veterinary Research Institute over a 5‐year period (2012–2016) showed that seroprevalence was lowest in goats (0.42%) and highest in buffaloes (0.99%). Although the number of buffaloes tested was relatively small, annual prevalence varied from 0% in buffaloes in 2015 to a peak of 1.65% in cattle in 2016. The mean annual prevalence over the study period was 0.75% [54]. In a study of randomly selected small ruminant farms in Selangor, the seroprevalence of melioidosis, determined using the complement fixation test, was 1% in goats and 0% in sheep [55]. A recent case report from Selangor state documented a Savanna goat infected with *B. pseudomallei*, with soil disturbance during excavation suspected as the possible source of infection [56]. Compared with other MSEA countries, Peninsular Malaysia has the most comprehensive veterinary surveillance data, providing stronger evidence for the wide host range of *B. pseudomallei*. However, differences in surveillance intensity limit direct comparisons with neighboring countries.

### 4.5. Thailand

In Thailand, outbreaks of animal melioidosis have been documented in both zoos and pig farms [47, 57]. A seroprevalence study among long‐tailed macaques from Kosumpee Forest Park in Maha Sarakham Province reported a rate of 57.14% [58]. More recently, exposure to *B. pseudomallei* has also been reported in dogs in southern Thailand, with a seroprevalence of 5.77%, potentially reflecting environmental exposure through contaminated soil and water sources [59]. Among livestock, the highest incidence of melioidosis was observed in goats (1.63 cases per 100,000), followed by pigs (0.02 per 100,000), and cattle (0.01 per 100,000) [60]. Post‐mortem examinations of fatal goat cases revealed multisystem involvement, with multiple abscesses commonly found in the lungs, liver, and spleen. Additionally, culture‐positive milk samples indicated mastitis, suggesting the potential for transmission of *B. pseudomallei* through goat milk [61]. The diversity of affected animal species and documented outbreaks indicate that melioidosis is well established in animals in Thailand, although the epidemiological importance of different animal hosts in maintaining transmission remains incompletely understood.

### 4.6. Vietnam

In Vietnam, *B. pseudomallei* has been identified in several animal species, including rats, pigs, rabbits, guinea pigs, and dogs [62]. More recently, in 2020, Norris et al. [63] published data indicating an average seroprevalence of exposure to *B. pseudomallei* in swine across three provinces (Ha Tinh, Hoa Binh, and Nghe An), with a rate of 6.3%. Current evidence suggests that animal exposure occurs in multiple provinces, but available data remain limited and are insufficient to define the national burden of animal melioidosis.

Overall, *B. pseudomallei* infection has been documented in various animal species across MSEA: it is well established in Peninsular Malaysia and Thailand, increasingly reported in Lao PDR and Vietnam, rarely reported in Cambodia, and not yet reported in Myanmar. This pattern likely reflects differences in veterinary surveillance and diagnostic capacity rather than the true distribution of the disease. Given that melioidosis is predominantly acquired from the environment, these findings primarily highlight the need to better characterize environmental exposure and infection in animal populations. Strengthening One Health surveillance would provide a more comprehensive understanding of the epidemiology of *B. pseudomallei* across the MSEA.

## 5. Human Melioidosis

Melioidosis is recognized as endemic throughout Southeast Asia. The global burden of human melioidosis has been estimated at 4.64 million disability‐adjusted life‐years (DALYs) per year, which is higher than that of any other officially recognized neglected and re‐emerging tropical disease estimated by WHO. In this assessment, among the six MSEA countries, Cambodia had the highest estimated DALY burden, at 415 DALYs per 100,000 [64]. Although melioidosis has a significant impact and was reported in Myanmar as early as 1911, it remains a non‐notifiable disease in MSEA, except in Thailand. Human cases have been reported in all six MSEA countries. However, differences in reported incidence between countries are strongly influenced by variations in surveillance systems, diagnostic access, microbiology laboratory capacity, healthcare access, and reporting practices and therefore should not be interpreted as reflecting true differences in disease burden (Table 2). Consequently, the incidence of melioidosis is likely underestimated in countries with limited diagnostic and surveillance capacity.

**Table 2:** Epidemiological information on human melioidosis in MSEA.

Country	Level of evidence	First report	Notifiable disease	Reference
Cambodia	2592 culture‐confirmed cases between 2005 and 2017 from 23 of 25 provinces.	1928	No	[65, 66]
Lao PDR	1744 human cases confirmed from 1999 to 2020.	1954	No	[49, 67, 68]
Myanmar	298 cases were reported in seven states from 1911 to 2017.	1911	No	[51, 69]
Peninsular Malaysia	6302 culture‐confirmed cases between 2014 and 2020.	1917	No (except in some states)	[52, 70, 71]
Thailand	4407 culture‐confirmed cases and 1219 deaths in 2022.	1955	Yes	[72–74]
Vietnam	55 culture‐confirmed cases from at least 18 northern provinces between 1997 and 2005, 70 cases in the north‐central region over a 7‐month period in 2015, and 35 culture‐confirmed pediatric cases from 13 provinces and cities between 2015 and 2019.	1925	No	[62, 75–78]

### 5.1. Cambodia

Melioidosis was first diagnosed in Cambodia in 1928 [65]. Between 2005 and 2017, a total of 2592 culture‐confirmed cases were reported, with at least 60% occurring in children. Culture‐confirmed *B. pseudomallei* was detected in 23 out of Cambodia’s 25 provinces, indicating that melioidosis is likely endemic across the country [66]. The estimated incidence ranged from 28 to 35 cases per 100,000 children, with a high mortality rate of 71.8% among bacteraemic cases [79]. Although melioidosis is not currently classified as a notifiable disease in Cambodia, *B. pseudomallei* has been designated as one of seven priority pathogens under the Ministry of Health’s Antimicrobial Resistance (AMR) surveillance program. Since 2018, this program has been monitoring phenotypic AMR patterns in eight tertiary hospitals across the country [80]. In addition, a retrospective serological study using serum samples from three studies conducted across 10 provinces reported a 12% seroprevalence of antibodies against *B. pseudomallei*, providing evidence of exposure to the bacterium among both patients with febrile illness and healthy individuals [81]. Among children attending a hospital in Siem Reap, Cambodia, 6.5% (63/968) had antibody titers ≥1:160 against *B. pseudomallei* by IHA, further supporting evidence of exposure to the pathogen [13]. The wide geographic distribution of confirmed cases and serological evidence of exposure indicate that melioidosis is endemic in Cambodia; however, the absence of mandatory national notification likely results in underestimation of its true burden.

### 5.2. Lao PDR

The earliest recorded cases of melioidosis in Lao PDR occurred during military operations in 1954 [67]. Melioidosis is not recognized as a notifiable disease in the country, and there is currently no formal surveillance system in place for human cases [49]. Between 1999 and 2020, a total of 1744 confirmed human cases were reported nationwide [68]. However, based on clinical experience at Mahosot Hospital in Vientiane, it is believed that hundreds of additional melioidosis‐related deaths occur annually, remaining undiagnosed and unreported [49]. The discrepancy between reported cases and clinical experience suggests that the burden of melioidosis in Lao PDR is substantially underestimated because of the limited surveillance and diagnostic capacity.

### 5.3. Myanmar

Melioidosis was first identified in Myanmar in 1911 by Alfred Whitmore [69], a British pathologist, at Rangoon (now Yangon) General Hospital. Between 1911 and 2018, a total of 298 cases were reported across seven states and regions, with the vast majority occurring before 1949. Currently, melioidosis is not a notifiable disease in Myanmar [51]. In a study conducted at four tertiary hospitals in Yangon between June 2017 and May 2019, *B. pseudomallei* was culture‐confirmed in 5.7% (21 out of 364) of patients [82]. Additionally, in a prospective cross‐sectional study of migrant workers from Myanmar in Thailand in 2005, the seroprevalence of exposure to *B. pseudomallei* by IHA was 7% with titers ≥1:160 [83]. More recently, serological screening of 124 febrile patients attending mobile clinics in the delta region during the 2016 rainy season found that 3.2% were positive for *B. pseudomallei* using two serological methods, OPS and Hcp1 ELISAs, suggesting possible recent exposure to *B. pseudomallei* [84]. These findings indicate that melioidosis persists in Myanmar despite the relatively small number of reported cases, highlighting probable under‐recognition of the disease.

### 5.4. Peninsular Malaysia

Melioidosis was first documented in 1917, with two cases further characterized in 1923 [70, 85]. Although it is not classified as a notifiable disease at the national level, certain states such as Pahang and Sabah have implemented reporting systems due to the high number of diagnosed cases each year [52]. According to the National Surveillance for Antibiotic Resistance (NSAR), the average incidence rate across all states in Peninsular Malaysia was 3.39 per 100,000 population during the 2014–2020 period, with Pahang exhibiting the highest rate at 11.33 per 100,000 [71]. A nationwide study conducted from 2015 to 2019 assessed seropositivity to *B. pseudomallei* patients admitted to healthcare centers across Malaysia. Analysis of 26,665 serum samples revealed a national seropositivity rate of 16.4%, with a rate of 13.9% specifically in Pahang [86]. Additionally, a cross‐sectional study from 2013 to 2014 analyzing 17,234 samples found an overall seropositivity rate of 10.5% using a titer cut‐off of 1:160. Notably, pediatric cases (children under 15 years of age) showed higher exposure to *B. pseudomallei* [87]. The higher incidence reported in states such as Pahang likely reflects a combination of true endemicity and greater diagnostic awareness, although nationwide surveillance remains incomplete.

### 5.5. Thailand

Melioidosis was first described in Thailand in 1955 [72]. Based on evidence collected from 1910 to 2015, the disease is likely endemic throughout the country, with the northeastern region identified as a hyperendemic area. Since 2002, melioidosis has been a notifiable disease in Thailand and must be reported under the National Notifiable Diseases Surveillance System (NNDSS) [74]. In 2022, 4407 culture‐confirmed melioidosis cases were reported, resulting in 1219 deaths [73]. A spatio‐temporal analysis conducted in Ubon Ratchathani Province (Northeast Thailand) from 2016 to 2020 revealed a declining trend in incidence, with the highest rate recorded in 2016 at 32.37 cases per 100,000 population, dropping to 10.83 cases per 100,000 by 2020 [88]. The monthly incidence data showed that melioidosis cases occurred across all seasons during the study period [88]. A spatiotemporal interpolation revealed high heterogeneity in melioidosis morbidity across sub‐districts, with hotspots primarily located in the northern areas of the province. The monthly morbidity rate was highest in January, followed by August and September [89]. In southern Thailand, the provinces of Songkhla and Phatthalung are considered seasonally endemic for melioidosis, with an average annual incidence of 2.87 cases per 100,000 population. Despite the lower incidence compared to the Northeast, the mortality rate remains similarly high in these southern provinces [40].

Following the 2004 tsunami in southern Thailand, 13.5% (7/52) of individuals directly involved in the tsunami had IHA titers ≥1:160 against *B. pseudomallei*, compared with 7.3% (76/1039) among individuals not documented as physically injured. This finding suggested a possible increase in *B. pseudomallei* exposure among individuals directly affected by the tsunami [45]. Additionally, a study of children admitted to hospitals in Udon Thani and Ubon Ratchathani (northeastern region), estimated annual antibody‐producing exposure rates of 24% and 11%, respectively, suggesting greater exposure to *B. pseudomallei* in Udon Thani than in Ubon Ratchathani [90]. Thailand provides the most comprehensive clinical surveillance data in the MSEA because of its mandatory notification system, making it an important regional reference for assessing the reported burden and distribution of melioidosis.

### 5.6. Vietnam

The first documented case of human melioidosis in Vietnam was reported in 1925 [78]. To date, melioidosis is not classified as a notifiable disease in the country [62]. A study conducted between November 1997 and December 2005 revealed that 55 culture‐confirmed cases were geographically distributed across at least 18 northern provinces, indicating a broad geographic distribution of reported cases [76]. In another study, *B. pseudomallei* was identified in 70 cases over a 7‐month period in 2015, with 82.8% of patients presenting with bacteremia. Among those with known outcomes, the mortality rate was 50% [75]. More recently, a retrospective observational study of 35 pediatric melioidosis cases demonstrated a wide geographic distribution across 13 of 32 provinces and cities, covering four regions of southern Vietnam: the southeast, south‐central coast, central highlands, and the Mekong River Delta [77]. An earlier study on the seroprevalence since 1993 in 4 communes in the northern part of Vietnam among a random sample of 406 people showed seropositivity ranging from 6.4% to 31.8% [91]. The broad geographic distribution of both clinical cases and serological evidence suggests that melioidosis is more widespread than routine case reporting indicates.

Across the MSEA countries, the seroprevalence of *B. pseudomallei* has been reported to vary widely, ranging from 3.2% (Myanmar) to 65.7% (Malaysia) depending on the detection method used and the targeted population (suspected cases, culture‐positive, febrile patients…) [92]. These include enzyme‐linked immunosorbent assay (ELISA), indirect hemagglutination assay (IHA), and immunofluorescent assay (IFA) [92].

In summary, the available evidence indicates that melioidosis is endemic throughout MSEA, but its regional epidemiology remains incompletely characterized. Countries with established surveillance systems, such as Thailand, report substantially higher numbers of confirmed cases than neighboring countries, yet this probably reflects greater case detection rather than a fundamentally higher disease burden. The widespread environmental distribution of *B. pseudomallei*, together with serological evidence of human exposure in all six countries, suggests that melioidosis may be underdiagnosed across the MSEA.

## 6. Risk Factors, Clinical Presentation, and Diagnosis

### 6.1. Risk Factors

Most individuals exposed to *B. pseudomallei* do not develop melioidosis; however, certain underlying conditions that compromise innate and adaptive immune responses, particularly diabetes, are significant risk factors for infection [6]. The common risk factors between Thailand and Malaysia are water and soil exposure, diabetes, chronic renal failure, thalassemia, hepatitis, and cancer [92]. In Peninsular Malaysia, the central state of Negeri Sembilan has been associated with a higher melioidosis‐related mortality rate. Contributing factors include Bumiputra ethnicity, and underlying comorbidities such as diabetes mellitus and hypertension [93]. In Lao PDR, children were found to be nearly three times more likely than adults to acquire *B. pseudomallei* infection during periods of high humidity [39]. In Cambodia, bacteremia and pneumonia were identified as clinical factors associated with mortality among pediatric patients with melioidosis [94]. Importantly, *B. pseudomallei* can infect individuals across all age groups, including newborns, children, and adults [77, 95].

### 6.2. Clinical Presentation and Diagnosis

Melioidosis presents with a wide spectrum of clinical manifestations, ranging from mild localized infection to severe sepsis [3]. Patients often develop acute febrile illness with symptoms such as fever, headache, and general malaise, and pneumonia is the most common presentation, varying from mild respiratory symptoms to rapidly progressive necrotizing pneumonia. Localized infections may occur in skin or soft tissues, typically following minor injuries, and can form abscesses in organs such as the liver, spleen, or prostate. Chronic melioidosis can mimic tuberculosis, with prolonged fever, weight loss, and persistent cough. In severe cases, patients may develop bloodstream infection and septic shock, which carries a high mortality rate despite appropriate treatment. Melioidosis is therefore known for its diverse, sometimes subtle, and often severe clinical presentations [3]. The severity and outcome of the clinical presentation of melioidosis depend on the route of exposure to the organism, the presence or absence of risk factors, the bacterial load and strain, and the presence or absence of specific *B. pseudomallei* virulence genes [96]. In the pediatric populations across Cambodia, Thailand, and Vietnam, suppurative parotitis is the most common clinical manifestation [77, 94, 97]. Furthermore, a study of 1352 culture‐confirmed melioidosis cases from nine hospitals in northeastern Thailand found that bacteremia and septic shock were the predominant presentations in patients aged 15 and older [2]. While most patients who survive to discharge remain alive for 1 year, hospital readmission for various complications is common [2]. In rural Cambodia, 69% presented with primary pneumonia, 14% with skin or soft‐tissue infection, 14% with intraabdominal infection, and 3% with urinary tract infection [98].

Diagnosis of melioidosis is confirmed by isolating *B. pseudomallei* from clinical specimens such as blood, sputum, and urine. Depending on the clinical presentation, swabs, pus, and fluid from normally sterile sites may also be collected for testing [6]. The current gold standard for detection remains bacterial culture; however, misidentification is a concern, particularly among laboratory personnel unfamiliar with the organism. *B. pseudomallei* may be mistaken for *Burkholderia cepacia*, *Bacillus* spp., or *Pseudomonas* spp., or even dismissed as a contaminant [99]. While culture has a specificity of 100%, its sensitivity is relatively low at 60.2% [100]. Misidentification has been reported in both human and animal cases across the MSEA [48, 101].

To improve diagnostic accuracy, a latex agglutination assay using a monoclonal antibody specific to *B. pseudomallei* is used to confirm the identification of *B. pseudomallei* from bacterial isolates, demonstrating 95.1% sensitivity and 99.7% specificity. It is highly reproducible, simple to perform even by inexperienced laboratory personnel and does not require expensive or specialized equipment [102]. The Thailand melioidosis network strongly recommends that this assay be made routinely available in all microbiology laboratories across the country [74]. Molecular approaches, such as realtime polymerase chain reaction (qPCR), have also been developed for detecting *B. pseudomallei* in clinical specimens. A recent study in Thailand found that targeting the BPSS1187 gene provided greater sensitivity and specificity than the commonly used TTS1‐orf2 target [103]. Matrix‐assisted laser desorption ionization‐time of flight mass spectrometry (MALDI‐TOF MS) has emerged as a reliable, high‐throughput technique for bacterial identification. A study conducted in Khon Kaen, Thailand, demonstrated its potential for distinguishing *B. pseudomallei* in environmental samples, yielding results comparable to those of whole‐genome sequencing (WGS) [104].

Serological diagnosis remains challenging due to the lack of a standardized method. The IHA is used as a reference serodiagnostic test; however, it has low specificity and sensitivity [105]. Its utility is limited by a short shelf life and nonstandardized antigen preparations. Its sensitivity ranges from 56% to 70%, and specificity varies depending on background seroprevalence in the healthy population [105]. To address these limitations, newer antigen targets have been evaluated to improve the diagnostic accuracy. ELISA‐based studies in Thailand and Cambodia using O‐polysaccharide (OPS) as the target antigen have reported sensitivities of 71.4%–76.6% and specificities of 90.4%–90.9% (Table 3) [81, 105]. Additionally, three MSEA countries, Thailand, Vietnam, and Peninsular Malaysia have evaluated hemolysin coregulated protein 1 (Hcp1) as an ELISA target, yielding high sensitivity (83.0%–93.7%) and specificity (96.3%–100%) [106–108]. Both OPS and Hcp1 antigens have also been used for detecting antibodies against *B. pseudomallei* in swine and human populations in Vietnam and Myanmar [63, 84]. The comparative ELISA between OPS and Hcp1 antigens showed that Hcp1 had a significantly higher diagnostic sensitivity than OPS [107]. Moreover, the Hcp1 antigen has been evaluated as a target for a rapid immunochromatography test (ICT) in Thailand, showing potential for point‐of‐care (POC) diagnosis [109].

**Table 3:** Sensitivity and specificity of antigens for the serological diagnosis of human melioidosis in MSEA.

Antigen	Sensitivity	Specificity	Country	Reference
Hcp1 (ELISA)	83.0%–93.7%	96.3%–100%	Peninsular Malaysia, Thailand, and Vietnam	[106–108]
Hcp1 (ICT)	88.3%	86.1%	Thailand	[109]
OPS (ELISA)	71.4%–76.6%	90.4%–90.9%	Cambodia and Thailand	[81, 105]

In summary, melioidosis in MSEA is a diverse and potentially severe infection strongly influenced by environmental exposure and underlying health conditions. Its broad clinical presentation and diagnostic challenges highlight the need for improved detection, particularly through sensitive molecular and serological approaches.

## 7. Treatment and Antimicrobial Resistance

### 7.1. Treatment and Guidelines

The international treatment guideline for melioidosis recommends a biphasic therapy regimen. The intensive phase consists of at least 10–14 days of intravenous antibiotic administration aimed at preventing death from overwhelming sepsis. Recommended options include ceftazidime (50 mg/kg, up to 2 g every 6–8 h for uncomplicated cases) and meropenem (25 mg/kg, up to 1 g and 40 mg/kg, up to 2 g every 8 h for cases involving neuromelioidosis or persistent bacteremia) [110, 111]. This is followed by the eradication phase, which involves a minimum of 3 months of oral antibiotic therapy to eliminate residual bacteria and reduce the risk of relapse. Preferred agents include trimethoprim/sulfamethoxazole (8 mg/40 mg/kg, up to 320 mg/1600 mg every 12 h) or amoxicillin/clavulanic acid (20 mg/5 mg/kg, up to 1000 mg/250 mg every 8 h) [110, 111]. Adjunctive therapy is required in sepsis patients, including maintenance of blood pressure, adequate glycemic control, management of respiratory and acute renal failure, and drainage of abscesses where possible [111].

Among the six MSEA countries, Myanmar is still without national treatment guidelines specific to melioidosis; identified cases are managed on a case‐by‐case basis [51]. In contrast, Cambodia published an updated national guideline for melioidosis in 2020, developed with input from international experts [112]. Lao PDR released the fourth edition of its national treatment guideline in 2012; however, its recommendations are not fully aligned with the current international standards [49]. In Malaysia, clinical and public health guidelines for melioidosis exist only at the state level in Pahang (Peninsular Malaysia) and Sabah and were developed by the respective state health departments [113]. Thailand published a national melioidosis treatment guideline in 2021, led by the Department of Disease Control, Ministry of Public Health, with contributions from international melioidosis experts [114]. Vietnam’s Ministry of Health also issued national guidelines for the diagnosis and treatment of melioidosis in 2019 [115].

### 7.2. Antimicrobial Resistance

*B. pseudomallei* is intrinsically resistant to several classes of antibiotics, including aminoglycosides, rifamycins, penicillins, cephalosporins, and cationic peptides such as colistin [116]. Interestingly, certain clinical isolates from Thailand were susceptible to aminoglycosides and macrolides due to reduced or absent expression of the AmrAB‐OprA efflux pump [117]. Similarly, susceptible isolates from Malaysia belonged to sequence types (STs) 881 and 997 and harbored a nonsynonymous mutation in the *amrB* gene (which encodes a component of the AmrAB‐OprA efflux pump) [118]. These findings illustrate the genetic diversity of *B. pseudomallei* underlying unusual antimicrobial susceptibility phenotypes, but their clinical significance remains limited given their rarity.

Overall, resistance to first‐line antimicrobials used for melioidosis remains uncommon across MSEA, and current evidence does not indicate widespread treatment failure associated with AMR. A prospective multicenter study conducted in northeastern Thailand from 2015 to 2018 assessed antibiotic susceptibility and β‐lactam resistance in 1317 clinical *B. pseudomallei* isolates and reported a low prevalence of β‐lactam resistance. The authors reported resistance rates of 0.15% for ceftazidime (2/1304 isolates) and 0.08% for amoxicillin‐clavulanic acid (1/1304 isolates), while 0.15% (2/1304 isolates) of primary isolates were classified as “less susceptible” to meropenem [119].

Genomic studies have identified sequence types associated with resistance to specific antimicrobials. A systematic review and meta‐analysis of studies published between 1924 and 2023 analyzed 2737 *B. pseudomallei* isolates representing 729 distinct sequence types (STs) [120]. The analysis found that ST46 was the most frequently reported sequence type, particularly in Southeast Asia, appearing in 35% of the included studies, while ST51 was the only sequence type identified across human, animal, and environmental sources [120]. A study from Peninsular Malaysia analyzed environmental isolates from four states (Perak, Pahang, Selangor, and Negeri Sembilan) and veterinary isolates from Pulau Pinang (Penang). Among the isolates, 4.2% (1/24) of ST46 isolates were resistant to meropenem, while 2.2% (1/45) and 11.1% (5/45) of ST51 isolates were resistant to co‐amoxiclav and co‐trimoxazole, respectively [121]. Eighty‐four clinical isolates obtained from patients in tertiary government hospitals throughout Malaysia were analyzed. Among these, ST46 was identified in 10 isolates originating from four states and one federal territory in Peninsular Malaysia (Perlis, Pulau Pinang, Melaka, Johor, and Kuala Lumpur), while ST51 was identified in eight isolates from five states and one federal territory (Perak, Selangor, Negeri Sembilan, Melaka, Johor, and Kuala Lumpur). One ST46 isolate and one ST51 isolate were resistant to three antimicrobial agents: amoxicillin‐clavulanate, tetracycline, and trimethoprim‐sulfamethoxazole [122]. These observations indicate that resistance can occur in specific genetic backgrounds, but the available data do not suggest widespread dissemination of resistant isolates.

Resistance to trimethoprim‐sulfamethoxazole has also been reported in Vietnam (10.9%, 34/312 isolates) [123], Lao PDR (0.8%, 5/620) [124], while in Cambodia, one clinical isolate (ST196) was resistant to trimethoprim‐sulfamethoxazole, with an MIC greater than 32 mg/L, and another isolate (ST531) showed reduced inhibition zones for both amoxicillin‐clavulanic acid and ceftazidime [125]. However, differences in antimicrobial susceptibility testing methods, interpretive criteria, and study populations complicate direct comparisons of resistance frequencies among countries. Overall, the available evidence indicates that acquired resistance remains uncommon across most of MSEA, with no current evidence of widespread resistance compromising standard melioidosis treatment. Continued antimicrobial susceptibility surveillance remains important to detect emerging resistance and inform treatment recommendations.

In summary, treatment practices across MSEA generally follow established melioidosis management principles, but variations and limited AMR data highlight regional gaps. Continued susceptibility surveillance is needed to monitor emerging resistance and guide treatment strategies.

## 8. Conclusions and Perspectives

Melioidosis remains a non‐notifiable disease in several MSEA countries. Among MSEA countries, Thailand and Peninsular Malaysia have contributed the most comprehensive data on *B. pseudomallei* in humans, animals, and environmental sources. In contrast, research in Lao PDR has primarily focused on the environment. Despite sharing similar environmental conditions across MSEA, melioidosis is likely to be underdiagnosed and underreported in Cambodia, Lao PDR, Myanmar, and Vietnam because of differences in diagnostic and surveillance capacity. To address this, the implementation of a One Health approach across MSEA is essential. This integrated strategy should involve coordinated actions at the local, regional, and national levels across health, agriculture, and environmental sectors, alongside international collaborations. Such efforts are critical for accurately assessing melioidosis prevalence, guiding public health interventions, and ultimately reducing the transmission and mortality associated with the disease.

## Author Contributions

**Kimyeun Oeurn:** formal analysis, writing – original draft, writing – review and editing. **Vincent Herbreteau**: conceptualization, writing – original draft, writing – review and editing. **Sokleaph Cheng**: writing – original draft. **Thi Thu Hang Le**: writing – original draft. **Anne-Laure Bañuls:** writing –review and editing. **Mallorie Hide**: conceptualization, supervision, writing – original draft, writing – review and editing.

## Funding

This work was supported by the AfriCam project, funded by the French Development Agency (AFD) as part of PREACTS (PREZODE in action in the global South) program.

## Disclosure

All AI‐assisted, ChatGPT (OpenAI) and DeepL, suggestions were reviewed by the authors, who take full responsibility for the final content of the manuscript.

## Conflicts of Interest

The authors declare no conflicts of interest.

## Data Availability

The authors have nothing to report.
